# Crystal structure and Hirshfeld surface analysis of two 5,11-methano­benzo[*g*][1,2,4]triazolo[1,5-*c*][1,3,5]oxa­diazo­cine derivatives

**DOI:** 10.1107/S2056989019003700

**Published:** 2019-03-26

**Authors:** Mustafa Kemal Gumus, Sevgi Kansiz, Cigdem Yuksektepe Ataol, Necmi Dege, Igor O. Fritsky

**Affiliations:** a Artvin Coruh University, Science-Technology Research and Application Center, Artvin 08000, Turkey; b Ondokuz Mayıs University, Faculty of Arts and Sciences, Department of Physics, 55139 Kurupelit, Samsun, Turkey; c Cankiri Karatekin University, Faculty of Science, Department of Physics, 18100 Cankiri, Turkey; d Taras Shevchenko National University of Kyiv, Department of Chemistry, 64 Vladimirska Str., Kiev 01601, Ukraine

**Keywords:** crystal structure, Biginelli condensation, benzoxa­diazo­cine, hydrogen bonding, C—H⋯π inter­actions, C—Br⋯π inter­actions, Hirshfeld surface analysis

## Abstract

In the title compounds, 9-bromo-2,5-dimethyl-11,12-di­hydro-5*H*-5,11-methano­benzo[*g*][1,2,4]triazolo[1,5-*c*][1,3,5]oxa­diazo­cine, C_13_H_13_BrN_4_O (**I**), and 7-meth­oxy-5-methyl-2-(pyridin-4-yl)-11,12-di­hydro-5*H*-5,11-methano­benzo[*g*][1,2,4]tri­azolo[1,5-*c*][1,3,5]oxa­diazo­cine, C_18_H_17_N_5_O_2_ (**II**), the triazole ring is inclined to the benzene ring by 85.15 (9) and 76.98 (5)° in compounds **I** and **II**, respectively. In **II**, the pyridine ring is almost coplanar with the triazole ring, having a dihedral angle of 4.19 (8)°. In the crystal of **I**, pairs of N—H⋯N hydrogen bonds link the mol­ecules to form inversion dimers with an *R*_2_^2^(8) ring motif. The dimers are linked by C—H⋯π and C—Br⋯π inter­actions forming layers parallel to the *bc* plane. In the crystal of **II**, mol­ecules are linked by N—H⋯N and C—H⋯O hydrogen bonds forming chains propagating along the *b*-axis direction. The inter­molecular inter­actions were investigated using Hirshfeld surface analysis and two-dimensional fingerprint plots, and the mol­ecular electrostatic potential surface was also analysed. The Hirshfeld surface analysis of **I** suggests that the most significant contributions to the crystal packing are H⋯H (42.4%) and O⋯H/H⋯O (17.9%) contacts. For compound **II**, the H⋯H (48.5%), C⋯H/H⋯C (19.6%) and N⋯H/H⋯N (16.9%) inter­actions are the most important contributions.

## Abstract 2

In the crystals of 9-bromo-2,5-dimethyl-11,12-di­hydro-5*H*-5,11-methano­benzo[*g*][1,2,4]triazolo[1,5-*c*][1,3,5]oxa­diazo­cine (**I**) and 7-meth­oxy-5-methyl-2-(pyridin-4-yl)-11,12-di­hydro-5*H*-5,11-methano­benzo[*g*] [1,2,4]triazolo[1,5-*c*][1,3,5]oxa­diazo­cine (**II**), N—H⋯N hydrogen bonds link the mol­ecules to form inversion dimers in **I** and chains along the [010] direction in **II**.

## Chemical context   

In organic synthesis, a useful method to develop a chemical complexity from simple starting building blocks is the application of multicomponent reactions (MCRs) (Dömling *et al.*, 2012 ▸; Van der Heijden *et al.*, 2013 ▸). When amino­azoles having at least two non-equivalent reaction centres are used as building blocks , the method is generally characterized by ambiguous selectivity and different reaction outcomes (Murlykina *et al.*, 2018 ▸). According to Sedash *et al.*, Biginelli-like MCRs of 3-amino-1,2,4-triazole with aldehydes and α-carbonyl CH-acids may generate several types of heterocyclic products (Sedash *et al.*, 2012 ▸). The same starting compound with acetone and a 2-hy­droxy­benzaldehyde derivative under acidic conditions leads to the formation of different products (Gorobets *et al.*, 2010 ▸; Kondratiuk *et al.*, 2016 ▸; Gümüş *et al.*, 2017 ▸; Komykhov *et al.*, 2017 ▸).

Continuing our studies on the synthesis and crystal structure analyses of derivatives of a new type of oxygen-bridged Biginelli compound (Aydemir *et al.*, 2018 ▸; Gümüş *et al.*, 2017 ▸, 2018*a* ▸,*b* ▸), two new novel Biginelli-like assemblies of 3-amino-5-methyl-1,2,4-triazole/5-amino-3-(pyridin-4-yl)-1,2,4-triazole with acetone and 5-bromo­salicyl­aldehyde/*o*-vanillin have been developed to offer easy access to the title compounds, **I** and **II**, examples of this new class of heterocycles.
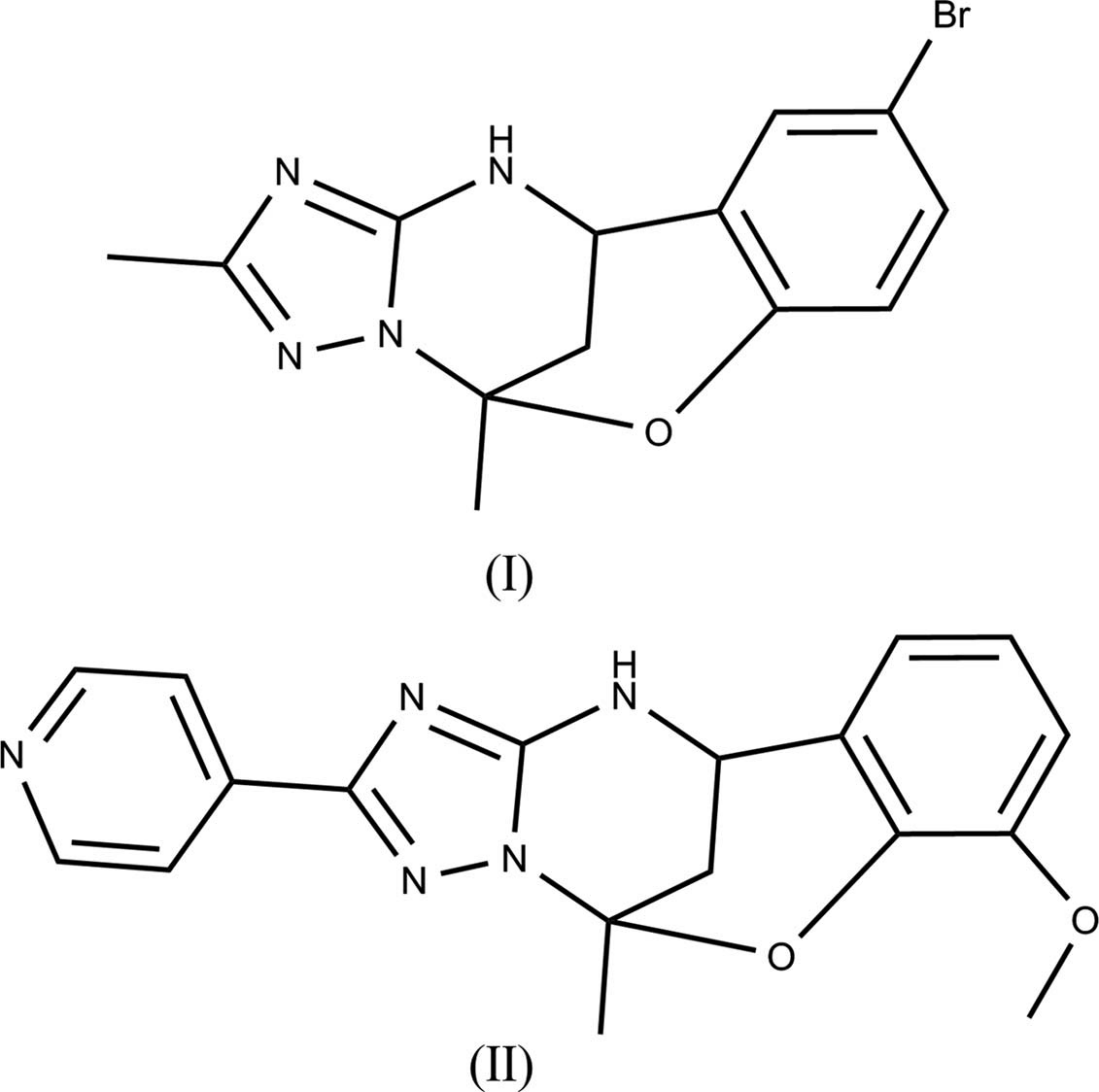


## Structural commentary   

**Figure 1:**
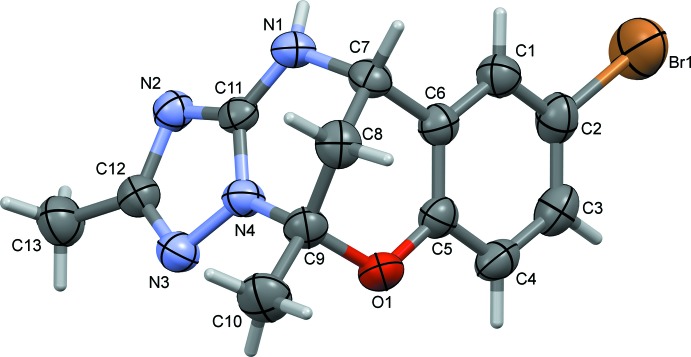
The mol­ecular structure of compound **I**, with the atom labelling. Displacement ellipsoids are drawn at the 50% probability level.

**Figure 3:**
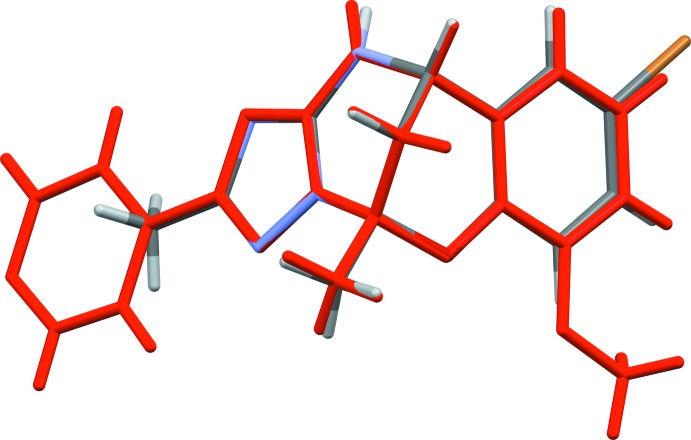
A view of the structural overlap of mol­ecules **I** and **II** (in red), having an r.m.s. deviation of 0.005 Å (*Mercury*; Macrae *et al.*, 2008 ▸).

The mol­ecular structures of compounds **I** and **II** are illustrated in Figs. 1 ▸ and 2 ▸, respectively. The conformations of the two compounds are very similar, as shown by the structural overlap of the two compounds [r.m.s. deviation = 0.005 Å (*Mercury*; Macrae *et al.*, 2008 ▸)], illustrated in Fig. 3 ▸. In **I**, the triazole ring (N2–N4/C11/C12) is inclined to the benzene ring (C1–C6) by 85.12 (12)°, compared to 76.96 (8)° in **II**. In the central 6-oxa-2,4λ^2^-di­aza­bicyclo­[3.3.1]nonane moiety, ring (N1/N4/C7–C9/C11) has a half-chair conformation in both compounds, while ring O1/C5–C9 has an envelope conformation, with atom C8 as the flap, in both compounds. The mean planes of these two rings are almost normal to each other, with a dihedral angle of 86.94 (11)° in **I** and 88.69 (8)° in **II**. In compound **II**, the pyridine ring (N5/C13–C17) is almost coplanar with the triazole ring, having a dihedral angle of 4.19 (8)°. The bond lengths and angles in the title compounds are very close to those observed for similar compounds, for example, the pyridin-3-yl analogue of compound **II** (Gümüş *et al.*, 2018 ▸); see also section *Database survey*.

## Supra­molecular features   

**Figure 4:**
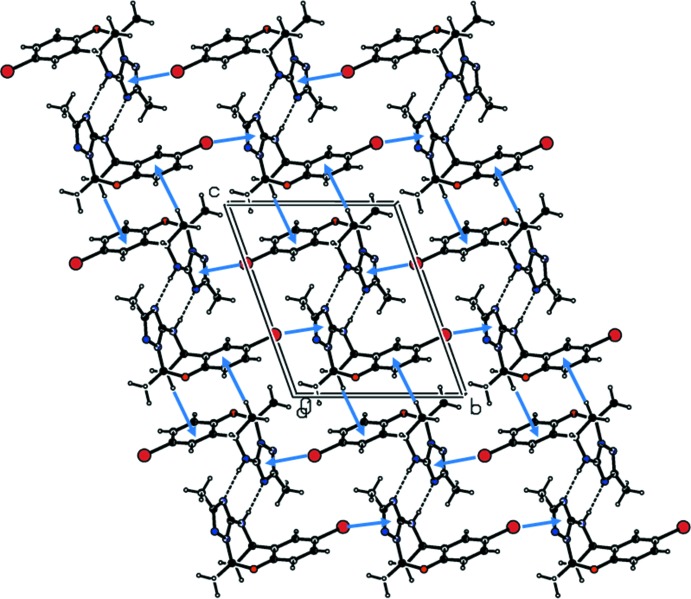
A view along the *a* axis of the crystal packing of compound **I**. Dashed lines denote the inter­molecular N—H⋯N hydrogen bonds, forming an inversion dimer with an 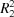
(8) ring motif (Table 1 ▸). C—H⋯π and C—Br⋯π inter­actions are shown as blue arrows (Table 1 ▸).

In the crystal of **I**, mol­ecules are linked by a pair of N—H⋯N hydrogen bonds, forming inversion dimers with an 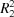
(8) ring motif (Table 1 ▸ and Fig. 4 ▸). The dimers are linked by C—H⋯π and C—Br⋯π inter­actions forming layers parallel to the *bc* plane (Table 1 ▸ and Fig. 4 ▸).

**Figure 5:**
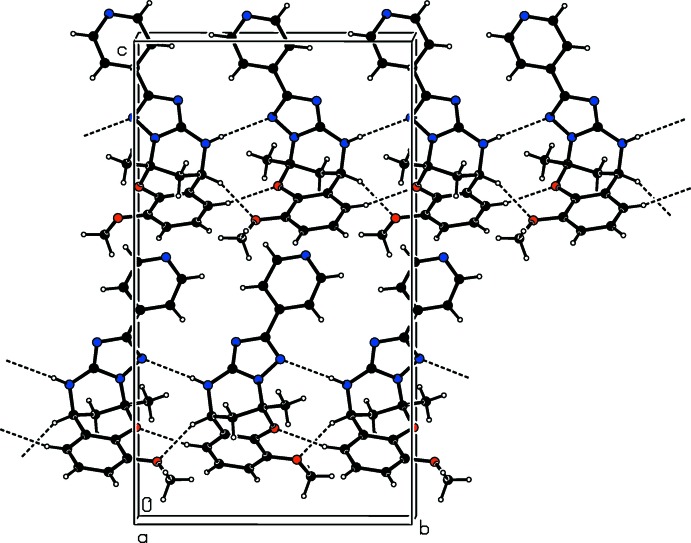
A view along the *a* axis of the crystal packing of compound **II**. Dashed lines denote inter­molecular hydrogen bonds (Table 2 ▸).

In the crystal of **II**, mol­ecules are connected *via* inter­molecular N—H⋯N and C—H⋯O hydrogen bonds, forming chains propagating along the *b*-axis direction (Table 2 ▸ and Fig. 5 ▸). Within the chains there are 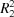
(10), 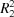
(11) and 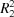
(9) ring motifs present (Table 2 ▸ and Fig. 5 ▸).

## Database survey   

A search of the Cambridge Structural Database (CSD, Version 5.40, update of November 2018; Groom *et al.*, 2016 ▸) for the triazolo-benzoxa­diazo­cine skeleton yielded 4 hits, namely 7-eth­oxy-5-methyl-11,12-di­hydro-5,11-methano­[1,2,4]triazolo[1,5-*c*][1,3,5]benzoxa­diazo­cine (HUVCEH; Gorobets *et al.*, 2010 ▸), 7-eth­oxy-5-methyl-2-(pyridin-3-yl)-11,12-di­hydro-5*H*-5,11-methano­[1,2,4]triazolo[1,5-*c*][1,3,5]benzoxa­diazo­cine (RETCAX; Aydemir *et al.*, 2018 ▸), 7-meth­oxy-5-methyl-2-phenyl-11,12-di­hydro-5*H*-5,11-methano­[1,2,4]triazolo[1,5-*c*][1,3,5]benzoxa­diazo­cine (SILBEX; Gümüş *et al.*, 2018*b* ▸), with two independent mol­ecules in the asymmetric unit, and 7-meth­oxy-5-methyl-2-(pyridin-3-yl)-11,12-di­hydro-5*H*-5,11-methano­[1,2,4]triazolo[1,5-*c*][1,3,5]benzoxa­diazo­cine (WEX­YUM; Gümüş *et al.*, 2018*a* ▸), also with two independent mol­ecules per asymmetric unit.

The conformations of all four compounds resemble those of compounds **I** and **II**, with the dihedral angle between the triazole and benzene rings varying from *ca* 71.20 to 87.37°, compared to 85.12 (12) and 76.96 (8)° in compounds **I** and **II**, respectively.

The geometrical parameters of the four compounds are very similar to each other and to those of compounds **I** and **II**. The C9—O1 and C5—O1 bond lengths are 1.456 (3) and 1.375 (3) Å, respectively, in **I** and 1.441 (2) and 1.385 (2) Å in **II**, compared to *ca* 1.445 and 1.374 Å in HUVCEH, 1.444 and 1.390 Å in RETCAX, 1.343/1.436 and 1.381/1.381 Å in SILBEX, and 1.429/1.444 and 1.377/1.380 Å in WEXYUW. In addition, the N3—N4 bond length is 1.388 (3) Å in **I** and 1.381 (2) Å in **II**, compared to *ca* 1.385, 1.389, 1.376/1.382 and 1.379/1.381 Å in HUVCEH, RETCAX, SILBEX and WEXYUW, respectively.

## Hirshfeld surface analysis   

**Figure 6:**
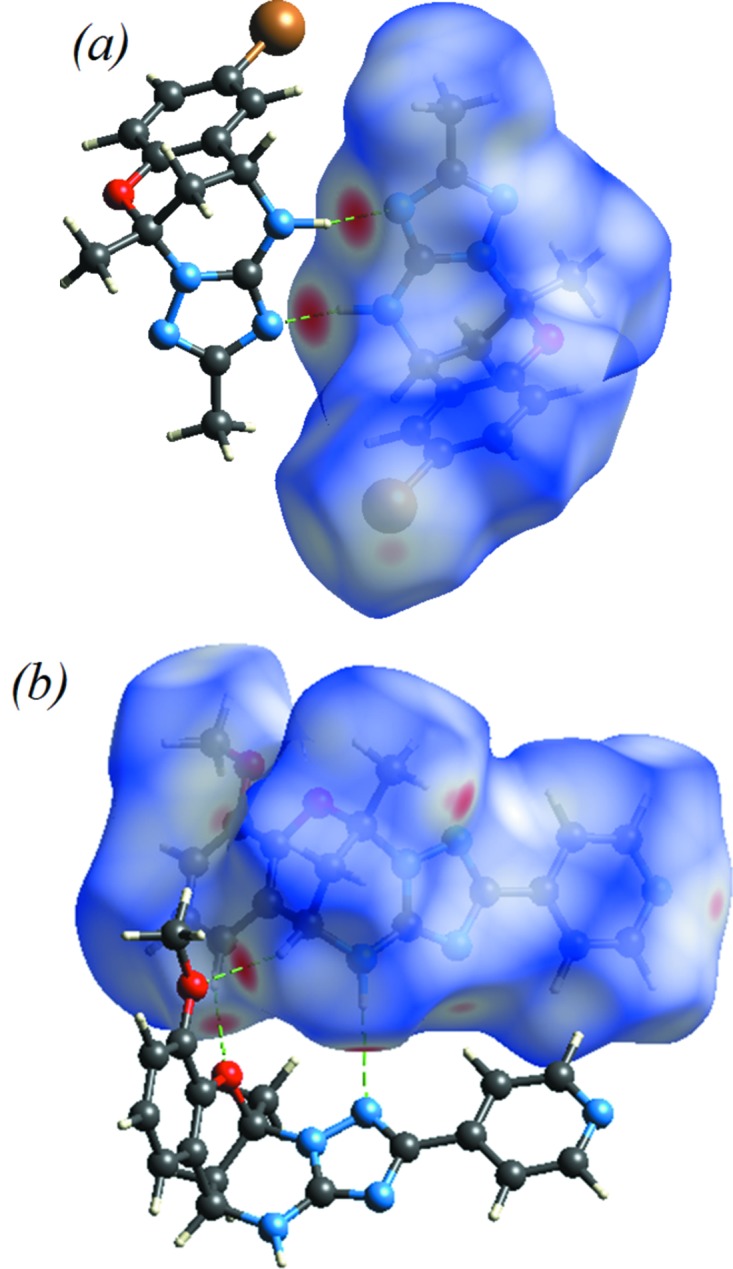
*d*_norm_ mapped on Hirshfeld surfaces for visualizing the inter­molecular inter­actions of (*a*) compound **I** and (*b*) compound **II**.

The Hirshfeld surface analysis (Spackman & Jayatilaka, 2009 ▸) and the associated two-dimensional (2D) fingerprint plots (McKinnon *et al.*, 2007 ▸) were performed with *CrystalExplorer17* (Turner *et al.*, 2017 ▸). The Hirshfeld surfaces were generated using a standard (high) surface resolution with the three-dimensional (3D) *d*_norm_ surfaces mapped over a fixed colour scale of −0.378 (red) to 1.282 Å (blue) for compound **I** and from −0.259 (red) to 1.216 Å (blue) for compound **II**. The red spots on the surface indicate the inter­molecular contacts involved in the hydrogen bonds. In Fig. 6 ▸(*a*), the identified red spot is attributed to the H⋯N close contacts. Also in Fig. 6 ▸(*a*), the N—H⋯N contacts are shown in the *d*_norm_ mapped surface as deep-red depression areas showing the inter­action between the neighbouring mol­ecules for compound **I**. Similarly, the red spots on the surface correspond to C—H⋯O and N—H⋯N hydrogen bonds in compound **II** [Chem scheme1] (Fig. 6 ▸ *b*).

**Figure 7:**
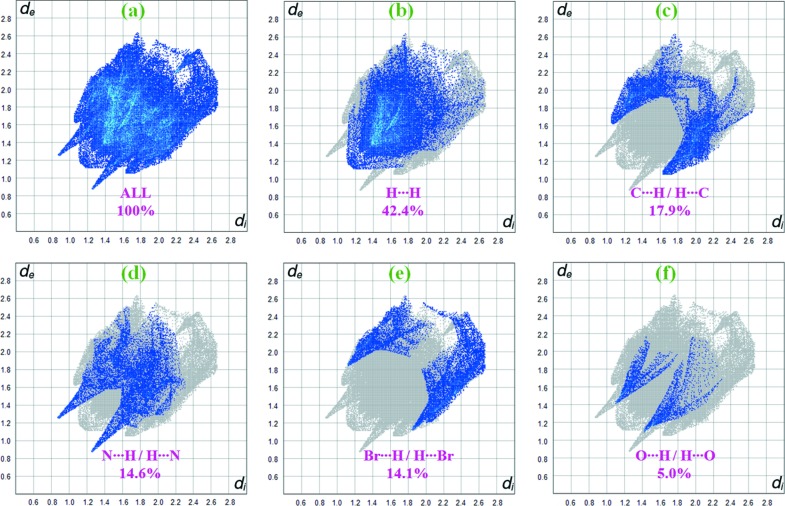
2D fingerprint plots for compound **I**, with a *d*_norm_ view and the relative contributions of the atom pairs to the Hirshfeld surface.

Fig. 7 ▸(*a*) shows the 2D fingerprint plot of the sum of the contacts contributing to the Hirshfeld surface of compound **I** represented in normal mode. 2D fingerprint plots provide information about the major and minor percentage contribution of the inter­atomic contacts in compound **I**. The blue colour refers to the frequency of occurrence of the (*d_i_*, *d_e_*) pair and the grey colour is the outline of the full fingerprint (Zaini *et al.*, 2019 ▸). The fingerprint plots (Fig. 7 ▸ *b*) show that the H⋯H contacts clearly make the most significant contribution to the Hirshfeld surface (42.4%). In addition, C⋯H/H⋯C, N⋯H/H⋯N and Br⋯H/H⋯Br contacts contribute 17.9, 14.6 and 14.1%, respectively, to the Hirshfeld surface. Much weaker O⋯H/H⋯O (5.0%), Br⋯N/N⋯Br (2.7%), Br⋯C/C⋯Br (1.8%) and Br⋯Br (1.0%) contacts also occur. In particular, the O⋯H/H⋯O contacts indicate the presence of inter­molecular C—H⋯O inter­actions.

**Figure 8:**
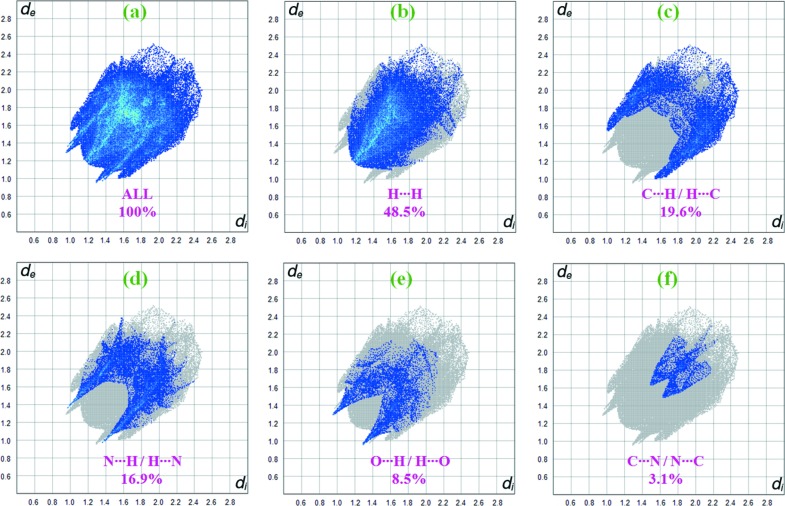
2D fingerprint plots for compound **II**, with a *d*_norm_ view and the relative contributions of the atom pairs to the Hirshfeld surface.

Similarly, for compound **II**, the H⋯H inter­actions appear in the middle of the scattered points in the 2D fingerprint plots with a contribution to the overall Hirshfeld surface of 48.5% (Fig. 8 ▸ *b*). The contribution from the N⋯H/H⋯N contacts, corresponding to the N—H⋯N inter­actions, is represented by a pair of sharp spikes characteristic of a strong hydrogen-bond inter­action (16.9%) (Fig. 8 ▸ *d*). The whole fingerprint region and all other inter­actions are displayed in Fig. 8 ▸.

**Figure 9:**
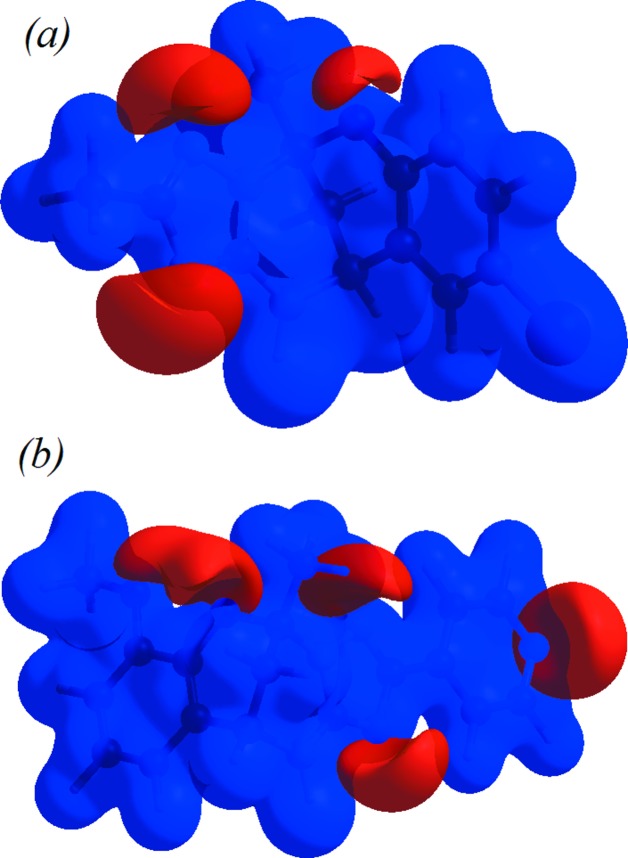
The view of the three-dimensional Hirshfeld surface of (*a*) compound **I** and (*b*) compound **II**, plotted over the electrostatic potential surface.

Views of the mol­ecular electrostatic potential, in the range −0.0500 to 0.0500 a.u. using the STO-3G basis set at the Hartree–Fock level of theory, for compounds **I** and **II** are shown in Figs. 9 ▸(*a*) and 9(*b*), respectively. In Fig. 9 ▸(*a*), the N—H⋯N hydrogen-bond donors and acceptors are shown as blue and red areas around the atoms related with positive (hydrogen-bond donors) and negative (hydrogen-bond acceptors) electrostatic potentials, respectively. Also, in Figs. 9 ▸(*a*) and 9(*b*), the N—H⋯N and C—H⋯O contacts in compounds **I** and **II** are given in the mol­ecular electrostatic potential mapped surface showing the inter­action between neighbouring mol­ecules.

## Synthesis and crystallization   

**Figure 10:**
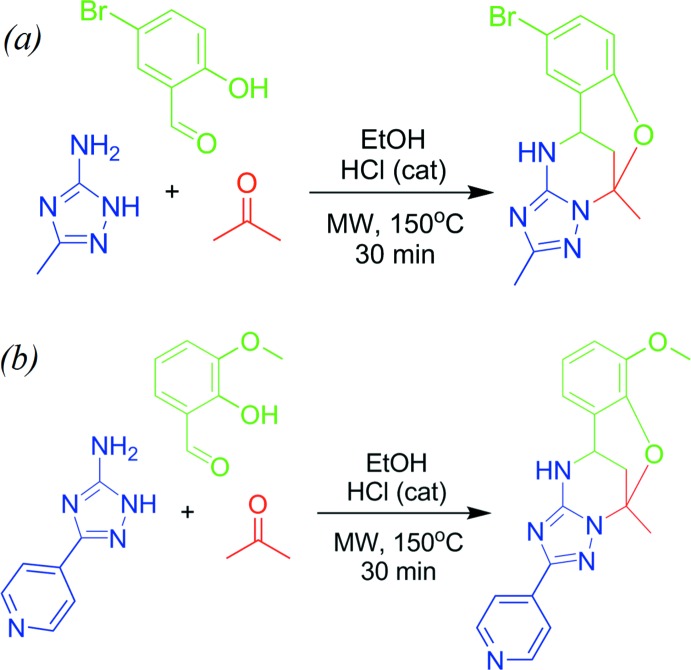
The synthesis of (*a*) compound **I** and (*b*) compound **II**.

The synthesis of the title compounds (Fig. 10 ▸) has been described by Gümüş *et al.* (2017 ▸). 3-Amino-5-methyl-1,2,4-triazole/3-amino-5-(pyridin-4-yl)-1,2,4-triazole (1.0 mmol), 5-bromo­salicyl­aldehyde (1.0 mmol) for compound **I** [*o*-vanillin (1.0 mmol) for compound **II**], acetone (0.22 ml, 3.0 mmol), and absolute EtOH (2.0 ml) were mixed in a microwave process vial, then a 4 *N* solution of HCl in dioxane (0.07 ml, 0.3 mmol) was added. The mixtures were irradiated at 423 K for 30 min. The reaction mixtures were cooled by an air flow and stirred for 24 h at room temperature for complete precipitation of the products. The precipitates were filtered off, washed with EtOH (1.0 ml) and Et_2_O (3 × 1.0 ml), and then dried. The compounds were obtained in the form of white solids. They were recrystallized from ethanol yielding colourless prismatic crystals for both compounds **I** and **II**.

## Refinement   

Crystal data, data collection and structure refinement details are summarized in Table 3 ▸. For compound **I**, the nitro­gen-bound H atom was located in a difference Fourier map and refined subject to a restraint of N—H = 0.86 (2) Å, while for compound **II**, the nitro­gen-bound H atom was also located in a difference Fourier map and was freely refined. For both compounds, the C-bound H atoms were positioned geom­etrically and refined using a riding model, with C—H = 0.93–0.97 Å and *U*_iso_(H) = 1.5*U*_eq_(C) for methyl H atoms and 1.2*U*_eq_(C) otherwise.

## Supplementary Material

Crystal structure: contains datablock(s) I, II, Global. DOI: 10.1107/S2056989019003700/su5490sup1.cif

Structure factors: contains datablock(s) I. DOI: 10.1107/S2056989019003700/su5490Isup2.hkl

Structure factors: contains datablock(s) II. DOI: 10.1107/S2056989019003700/su5490IIsup3.hkl

CCDC references: 1903781, 1882558

Additional supporting information:  crystallographic information; 3D view; checkCIF report

## Figures and Tables

**Figure 2:**
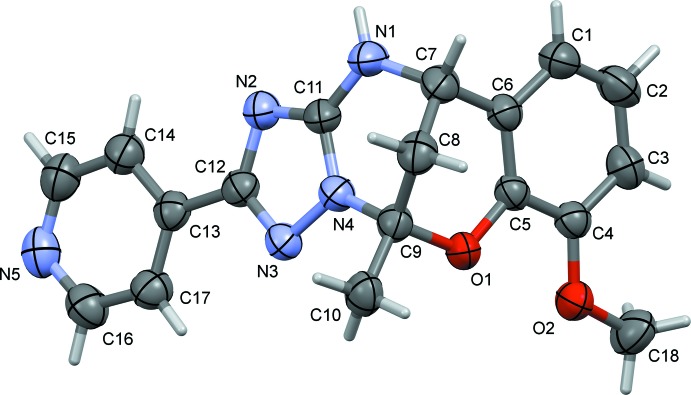
The mol­ecular structure of compound **II**, with the atom labelling. Displacement ellipsoids are drawn at the 50% probability level.

**Table 1:** Hydrogen-bond geometry (Å, °) for (I) *Cg*1 and *Cg*4 are the centroids of rings N2–N4/C11/C12 and C1–C6.

*D*—H⋯*A*	*D*—H	H⋯*A*	*D*⋯*A*	*D*—H⋯*A*
N1—H1*A*⋯N2^i^	0.83 (2)	2.13 (2)	2.949 (3)	174 (2)
C8—H8*A*⋯*Cg*4^ii^	0.97	2.86	3.823 (3)	175
C2—Br1⋯*Cg*1^iii^	1.89 (1)	3.40 (1)	4.724 (3)	124 (1)

Symmetry codes: (i) 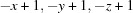; (ii) 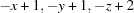; (iii) 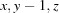.

**Table 2:** Hydrogen-bond geometry (Å, °) for (II)

*D*—H⋯*A*	*D*—H	H⋯*A*	*D*⋯*A*	*D*—H⋯*A*
N1—H1*A*⋯N3^i^	0.83 (2)	2.53 (2)	3.356 (2)	169 (2)
C1—H1⋯O1^i^	0.93	2.53	3.426 (2)	163
C7—H7⋯O2^i^	0.98	2.35	3.264 (2)	155

Symmetry code: (i) 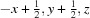.

**Table 3:** Experimental details

	(I)	(II)
Crystal data		
Chemical formula	C_13_H_13_BrN_4_O	C_18_H_17_N_5_O_2_
*M*_r_	321.18	335.36
Crystal system, space group	Triclinic, *P* 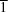	Orthorhombic, *P* *b* *c* *a*
Temperature (K)	296	296
*a*, *b*, *c* (Å)	6.1446 (6), 9.7407 (8), 11.6801 (11)	11.2814 (6), 12.6299 (6), 22.0008 (15)
α, β, γ (°)	109.657 (7), 92.325 (8), 91.664 (7)	90, 90, 90
*V* (Å^3^)	657.13 (11)	3134.7 (3)
*Z*	2	8
Radiation type	Mo *K*α	Mo *K*α
μ (mm^−1^)	3.13	0.10
Crystal size (mm)	0.34 × 0.19 × 0.11	0.31 × 0.22 × 0.15

Data collection		
Diffractometer	Stoe IPDS 2	Stoe IPDS 2
Absorption correction	Integration (*X-RED32*; Stoe & Cie, 2002 ▸)	Integration (*X-RED32*; Stoe & Cie, 2002 ▸)
*T*_min_, *T*_max_	0.477, 0.748	0.975, 0.986
No. of measured, independent and observed [*I* > 2σ(*I*)] reflections	12939, 4242, 2223	23575, 4432, 1789
*R*_int_	0.051	0.086
(sin θ/λ)_max_ (Å^−1^)	0.729	0.698

Refinement		
*R*[*F* ^2^ > 2σ(*F* ^2^)], *wR*(*F* ^2^), *S*	0.045, 0.115, 0.98	0.039, 0.073, 0.80
No. of reflections	4242	4432
No. of parameters	178	231
No. of restraints	1	0
H-atom treatment	H atoms treated by a mixture of independent and constrained refinement	H atoms treated by a mixture of independent and constrained refinement
Δρ_max_, Δρ_min_ (e Å^−3^)	0.28, −0.70	0.16, −0.14

Computer programs: *X-AREA* and *X-RED32* (Stoe & Cie, 2002 ▸), *SHELXT2017* (Sheldrick, 2015*a* ▸), *Mercury* (Macrae *et al.*, 2008 ▸), *WinGX* (Farrugia, 2012 ▸), *SHELXL2018* (Sheldrick, 2015*b* ▸), *PLATON* (Spek, 2009 ▸) and *publCIF* (Westrip, 2010 ▸).

## supplementary crystallographic information

### 9-Bromo-2,5-dimethyl-11,12-dihydro-5H-5,11-methanobenzo[g][1,2,4]triazolo[1,5-c][1,3,5]oxadiazocine (I) Crystal data

**Table:**

C_13_H_13_BrN_4_O	*Z* = 2
*M**_r_* = 321.18	*F*(000) = 324
Triclinic, *P*1	*D*_x_ = 1.623 Mg m^−^^3^
*a* = 6.1446 (6) Å	Mo *K*α radiation, λ = 0.71073 Å
*b* = 9.7407 (8) Å	Cell parameters from 9169 reflections
*c* = 11.6801 (11) Å	θ = 2.4–31.5°
α = 109.657 (7)°	µ = 3.13 mm^−^^1^
β = 92.325 (8)°	*T* = 296 K
γ = 91.664 (7)°	Prism, colourless
*V* = 657.13 (11) Å^3^	0.34 × 0.19 × 0.11 mm

### 9-Bromo-2,5-dimethyl-11,12-dihydro-5H-5,11-methanobenzo[g][1,2,4]triazolo[1,5-c][1,3,5]oxadiazocine (I) Data collection

**Table:**

Stoe IPDS 2 diffractometer	4242 independent reflections
Radiation source: sealed X-ray tube, 12 x 0.4 mm long-fine focus	2223 reflections with *I* > 2σ(*I*)
Detector resolution: 6.67 pixels mm^-1^	*R*_int_ = 0.051
rotation method scans	θ_max_ = 31.2°, θ_min_ = 2.4°
Absorption correction: integration (X-RED32; Stoe & Cie, 2002)	*h* = −8→8
*T*_min_ = 0.477, *T*_max_ = 0.748	*k* = −14→13
12939 measured reflections	*l* = −16→16

### 9-Bromo-2,5-dimethyl-11,12-dihydro-5H-5,11-methanobenzo[g][1,2,4]triazolo[1,5-c][1,3,5]oxadiazocine (I) Refinement

**Table:**

Refinement on *F*^2^	Primary atom site location: structure-invariant direct methods
Least-squares matrix: full	Secondary atom site location: difference Fourier map
*R*[*F*^2^ > 2σ(*F*^2^)] = 0.045	Hydrogen site location: mixed
*wR*(*F*^2^) = 0.115	H atoms treated by a mixture of independent and constrained refinement
*S* = 0.98	*w* = 1/[σ^2^(*F*_o_^2^) + (0.0513*P*)^2^] where *P* = (*F*_o_^2^ + 2*F*_c_^2^)/3
4242 reflections	(Δ/σ)_max_ < 0.001
178 parameters	Δρ_max_ = 0.28 e Å^−^^3^
1 restraint	Δρ_min_ = −0.70 e Å^−^^3^

### 9-Bromo-2,5-dimethyl-11,12-dihydro-5H-5,11-methanobenzo[g][1,2,4]triazolo[1,5-c][1,3,5]oxadiazocine (I) Special details

**Table:**

Geometry. All esds (except the esd in the dihedral angle between two l.s. planes) are estimated using the full covariance matrix. The cell esds are taken into account individually in the estimation of esds in distances, angles and torsion angles; correlations between esds in cell parameters are only used when they are defined by crystal symmetry. An approximate (isotropic) treatment of cell esds is used for estimating esds involving l.s. planes.

### 9-Bromo-2,5-dimethyl-11,12-dihydro-5H-5,11-methanobenzo[g][1,2,4]triazolo[1,5-c][1,3,5]oxadiazocine (I) Fractional atomic coordinates and isotropic or equivalent isotropic displacement parameters (Å^2^)

**Table:**

	*x*	*y*	*z*	*U*_iso_*/*U*_eq_	
Br1	0.50800 (8)	−0.00902 (3)	0.68588 (4)	0.09386 (19)	
O1	0.1825 (3)	0.59496 (18)	0.89977 (14)	0.0477 (4)	
N1	0.5602 (3)	0.5822 (2)	0.66868 (17)	0.0408 (4)	
H1A	0.613 (4)	0.519 (2)	0.6116 (19)	0.041 (7)*	
N2	0.2699 (3)	0.63495 (19)	0.54765 (16)	0.0406 (4)	
N3	0.0498 (3)	0.7412 (2)	0.70145 (17)	0.0452 (4)	
N4	0.2379 (3)	0.69449 (19)	0.74411 (16)	0.0392 (4)	
C1	0.5355 (4)	0.2973 (3)	0.7486 (2)	0.0473 (5)	
H1	0.669578	0.280839	0.713372	0.057*	
C2	0.4059 (5)	0.1815 (3)	0.7523 (2)	0.0532 (6)	
C3	0.2065 (5)	0.2025 (3)	0.8038 (2)	0.0541 (6)	
H3	0.120166	0.123167	0.805244	0.065*	
C4	0.1360 (4)	0.3423 (3)	0.8533 (2)	0.0483 (6)	
H4	0.002492	0.357929	0.889189	0.058*	
C5	0.2649 (4)	0.4592 (2)	0.84915 (19)	0.0414 (5)	
C6	0.4666 (4)	0.4381 (2)	0.79711 (19)	0.0402 (5)	
C7	0.6010 (4)	0.5663 (2)	0.7867 (2)	0.0415 (5)	
H7	0.756370	0.552230	0.799071	0.050*	
C8	0.5347 (4)	0.7044 (3)	0.8840 (2)	0.0465 (5)	
H8A	0.576019	0.701721	0.964269	0.056*	
H8B	0.607448	0.789210	0.874330	0.056*	
C9	0.2895 (4)	0.7133 (2)	0.87046 (19)	0.0433 (5)	
C10	0.1984 (5)	0.8514 (3)	0.9542 (2)	0.0613 (7)	
H10A	0.042029	0.844207	0.945156	0.092*	
H10B	0.251676	0.933353	0.933786	0.092*	
H10C	0.243425	0.864266	1.037001	0.092*	
C11	0.3650 (4)	0.6328 (2)	0.65048 (19)	0.0355 (4)	
C12	0.0793 (4)	0.7029 (2)	0.5845 (2)	0.0437 (5)	
C13	−0.0777 (5)	0.7335 (3)	0.4974 (3)	0.0612 (7)	
H13A	−0.030509	0.821212	0.483641	0.092*	
H13B	−0.219248	0.745678	0.530443	0.092*	
H13C	−0.085106	0.653506	0.421778	0.092*	

### 9-Bromo-2,5-dimethyl-11,12-dihydro-5H-5,11-methanobenzo[g][1,2,4]triazolo[1,5-c][1,3,5]oxadiazocine (I) Atomic displacement parameters (Å^2^)

**Table:**

	*U*^11^	*U*^22^	*U*^33^	*U*^12^	*U*^13^	*U*^23^
Br1	0.1300 (4)	0.04262 (16)	0.1075 (3)	0.01035 (16)	0.0421 (2)	0.01877 (15)
O1	0.0489 (10)	0.0537 (9)	0.0436 (9)	0.0088 (7)	0.0140 (7)	0.0188 (7)
N1	0.0360 (10)	0.0454 (9)	0.0406 (10)	0.0051 (8)	0.0096 (8)	0.0129 (8)
N2	0.0446 (11)	0.0401 (9)	0.0375 (9)	0.0031 (8)	0.0050 (8)	0.0134 (7)
N3	0.0408 (11)	0.0513 (10)	0.0448 (11)	0.0092 (8)	0.0046 (9)	0.0170 (8)
N4	0.0369 (10)	0.0436 (9)	0.0364 (9)	0.0062 (8)	0.0065 (8)	0.0118 (7)
C1	0.0491 (14)	0.0480 (12)	0.0449 (13)	0.0068 (10)	0.0054 (10)	0.0152 (10)
C2	0.0699 (18)	0.0412 (11)	0.0495 (14)	0.0020 (11)	0.0044 (13)	0.0163 (10)
C3	0.0623 (17)	0.0528 (13)	0.0510 (14)	−0.0091 (12)	0.0021 (12)	0.0237 (11)
C4	0.0442 (14)	0.0637 (14)	0.0422 (12)	−0.0030 (11)	0.0054 (10)	0.0249 (11)
C5	0.0450 (13)	0.0485 (11)	0.0329 (10)	0.0042 (10)	0.0029 (9)	0.0164 (9)
C6	0.0393 (13)	0.0452 (11)	0.0363 (11)	0.0010 (9)	−0.0012 (9)	0.0145 (9)
C7	0.0326 (12)	0.0454 (11)	0.0458 (12)	0.0020 (9)	0.0009 (9)	0.0147 (9)
C8	0.0479 (14)	0.0454 (11)	0.0423 (12)	−0.0034 (10)	−0.0057 (10)	0.0111 (9)
C9	0.0495 (14)	0.0434 (11)	0.0350 (11)	0.0058 (10)	0.0063 (10)	0.0098 (9)
C10	0.0732 (19)	0.0571 (14)	0.0457 (14)	0.0131 (13)	0.0099 (13)	0.0051 (11)
C11	0.0351 (12)	0.0344 (9)	0.0367 (11)	−0.0019 (8)	0.0059 (9)	0.0114 (8)
C12	0.0441 (13)	0.0452 (11)	0.0433 (13)	0.0040 (10)	0.0045 (10)	0.0163 (9)
C13	0.0602 (18)	0.0716 (17)	0.0561 (15)	0.0146 (14)	0.0005 (13)	0.0267 (13)

### 9-Bromo-2,5-dimethyl-11,12-dihydro-5H-5,11-methanobenzo[g][1,2,4]triazolo[1,5-c][1,3,5]oxadiazocine (I) Geometric parameters (Å, º)

**Table:**

Br1—C2	1.892 (2)	C4—C5	1.383 (3)
O1—C5	1.375 (3)	C4—H4	0.9300
O1—C9	1.456 (3)	C5—C6	1.393 (3)
N1—C11	1.346 (3)	C6—C7	1.518 (3)
N1—C7	1.451 (3)	C7—C8	1.519 (3)
N1—H1A	0.825 (16)	C7—H7	0.9800
N2—C11	1.321 (3)	C8—C9	1.517 (4)
N2—C12	1.374 (3)	C8—H8A	0.9700
N3—C12	1.311 (3)	C8—H8B	0.9700
N3—N4	1.388 (3)	C9—C10	1.511 (3)
N4—C11	1.350 (3)	C10—H10A	0.9600
N4—C9	1.445 (3)	C10—H10B	0.9600
C1—C2	1.375 (4)	C10—H10C	0.9600
C1—C6	1.383 (3)	C12—C13	1.482 (4)
C1—H1	0.9300	C13—H13A	0.9600
C2—C3	1.377 (4)	C13—H13B	0.9600
C3—C4	1.378 (4)	C13—H13C	0.9600
C3—H3	0.9300		
			
C5—O1—C9	116.13 (17)	C8—C7—H7	109.7
C11—N1—C7	115.79 (18)	C9—C8—C7	108.11 (18)
C11—N1—H1A	119.1 (17)	C9—C8—H8A	110.1
C7—N1—H1A	114.7 (17)	C7—C8—H8A	110.1
C11—N2—C12	103.07 (18)	C9—C8—H8B	110.1
C12—N3—N4	101.80 (18)	C7—C8—H8B	110.1
C11—N4—N3	109.66 (17)	H8A—C8—H8B	108.4
C11—N4—C9	125.84 (19)	N4—C9—O1	109.02 (17)
N3—N4—C9	124.48 (18)	N4—C9—C10	111.4 (2)
C2—C1—C6	120.1 (2)	O1—C9—C10	105.3 (2)
C2—C1—H1	119.9	N4—C9—C8	106.70 (19)
C6—C1—H1	119.9	O1—C9—C8	109.36 (18)
C1—C2—C3	121.2 (2)	C10—C9—C8	114.9 (2)
C1—C2—Br1	118.6 (2)	C9—C10—H10A	109.5
C3—C2—Br1	120.20 (19)	C9—C10—H10B	109.5
C2—C3—C4	119.4 (2)	H10A—C10—H10B	109.5
C2—C3—H3	120.3	C9—C10—H10C	109.5
C4—C3—H3	120.3	H10A—C10—H10C	109.5
C3—C4—C5	119.7 (2)	H10B—C10—H10C	109.5
C3—C4—H4	120.2	N2—C11—N1	128.76 (19)
C5—C4—H4	120.2	N2—C11—N4	110.04 (19)
O1—C5—C4	116.1 (2)	N1—C11—N4	121.19 (19)
O1—C5—C6	122.9 (2)	N3—C12—N2	115.4 (2)
C4—C5—C6	121.0 (2)	N3—C12—C13	122.9 (2)
C1—C6—C5	118.6 (2)	N2—C12—C13	121.7 (2)
C1—C6—C7	120.8 (2)	C12—C13—H13A	109.5
C5—C6—C7	120.5 (2)	C12—C13—H13B	109.5
N1—C7—C6	110.99 (18)	H13A—C13—H13B	109.5
N1—C7—C8	108.26 (18)	C12—C13—H13C	109.5
C6—C7—C8	108.33 (19)	H13A—C13—H13C	109.5
N1—C7—H7	109.7	H13B—C13—H13C	109.5
C6—C7—H7	109.7		
			
C12—N3—N4—C11	0.2 (2)	C11—N4—C9—O1	−97.7 (2)
C12—N3—N4—C9	178.8 (2)	N3—N4—C9—O1	84.0 (2)
C6—C1—C2—C3	0.0 (4)	C11—N4—C9—C10	146.5 (2)
C6—C1—C2—Br1	−179.53 (18)	N3—N4—C9—C10	−31.8 (3)
C1—C2—C3—C4	−0.4 (4)	C11—N4—C9—C8	20.3 (3)
Br1—C2—C3—C4	179.14 (19)	N3—N4—C9—C8	−158.00 (19)
C2—C3—C4—C5	0.8 (4)	C5—O1—C9—N4	70.6 (2)
C9—O1—C5—C4	−165.8 (2)	C5—O1—C9—C10	−169.73 (19)
C9—O1—C5—C6	14.7 (3)	C5—O1—C9—C8	−45.7 (2)
C3—C4—C5—O1	179.6 (2)	C7—C8—C9—N4	−51.2 (2)
C3—C4—C5—C6	−0.8 (3)	C7—C8—C9—O1	66.6 (2)
C2—C1—C6—C5	0.0 (3)	C7—C8—C9—C10	−175.2 (2)
C2—C1—C6—C7	−176.2 (2)	C12—N2—C11—N1	−178.0 (2)
O1—C5—C6—C1	179.9 (2)	C12—N2—C11—N4	0.7 (2)
C4—C5—C6—C1	0.4 (3)	C7—N1—C11—N2	−167.4 (2)
O1—C5—C6—C7	−3.9 (3)	C7—N1—C11—N4	14.1 (3)
C4—C5—C6—C7	176.6 (2)	N3—N4—C11—N2	−0.6 (2)
C11—N1—C7—C6	72.2 (2)	C9—N4—C11—N2	−179.14 (19)
C11—N1—C7—C8	−46.6 (2)	N3—N4—C11—N1	178.19 (18)
C1—C6—C7—N1	81.8 (3)	C9—N4—C11—N1	−0.4 (3)
C5—C6—C7—N1	−94.2 (2)	N4—N3—C12—N2	0.3 (3)
C1—C6—C7—C8	−159.5 (2)	N4—N3—C12—C13	−178.2 (2)
C5—C6—C7—C8	24.5 (3)	C11—N2—C12—N3	−0.6 (3)
N1—C7—C8—C9	66.4 (2)	C11—N2—C12—C13	177.9 (2)
C6—C7—C8—C9	−54.0 (2)		

### 9-Bromo-2,5-dimethyl-11,12-dihydro-5H-5,11-methanobenzo[g][1,2,4]triazolo[1,5-c][1,3,5]oxadiazocine (I) Hydrogen-bond geometry (Å, º)

Cg1 and Cg4 are the centroids of rings N2-N4/C11/C12 and C1-C6.

**Table:**

*D*—H···*A*	*D*—H	H···*A*	*D*···*A*	*D*—H···*A*
N1—H1*A*···N2^i^	0.83 (2)	2.13 (2)	2.949 (3)	174 (2)
C8—H8*A*···*Cg*4^ii^	0.97	2.86	3.823 (3)	175
C2—Br1···*Cg*1^iii^	1.89 (1)	3.40 (1)	4.724 (3)	124 (1)

Symmetry codes: (i) −*x*+1, −*y*+1, −*z*+1; (ii) −*x*+1, −*y*+1, −*z*+2; (iii) *x*, *y*−1, *z*.

### 7-Methoxy-5-methyl-2-(pyridin-4-yl)-11,12-dihydro-5H-5,11-methanobenzo[g][1,2,4]triazolo[1,5-c][1,3,5]oxadiazocine (II) Crystal data

**Table:**

C_18_H_17_N_5_O_2_	*D*_x_ = 1.421 Mg m^−^^3^
*M**_r_* = 335.36	Mo *K*α radiation, λ = 0.71073 Å
Orthorhombic, *P**b**c**a*	Cell parameters from 10886 reflections
*a* = 11.2814 (6) Å	θ = 1.6–30.1°
*b* = 12.6299 (6) Å	µ = 0.10 mm^−^^1^
*c* = 22.0008 (15) Å	*T* = 296 K
*V* = 3134.7 (3) Å^3^	Prisim, colorless
*Z* = 8	0.31 × 0.22 × 0.15 mm
*F*(000) = 1408	

### 7-Methoxy-5-methyl-2-(pyridin-4-yl)-11,12-dihydro-5H-5,11-methanobenzo[g][1,2,4]triazolo[1,5-c][1,3,5]oxadiazocine (II) Data collection

**Table:**

Stoe IPDS 2 diffractometer	4432 independent reflections
Radiation source: sealed X-ray tube, 12 x 0.4 mm long-fine focus	1789 reflections with *I* > 2σ(*I*)
Detector resolution: 6.67 pixels mm^-1^	*R*_int_ = 0.086
rotation method scans	θ_max_ = 29.7°, θ_min_ = 1.9°
Absorption correction: integration (X-RED32; Stoe & Cie, 2002)	*h* = −15→15
*T*_min_ = 0.975, *T*_max_ = 0.986	*k* = −15→17
23575 measured reflections	*l* = −30→29

### 7-Methoxy-5-methyl-2-(pyridin-4-yl)-11,12-dihydro-5H-5,11-methanobenzo[g][1,2,4]triazolo[1,5-c][1,3,5]oxadiazocine (II) Refinement

**Table:**

Refinement on *F*^2^	Primary atom site location: structure-invariant direct methods
Least-squares matrix: full	Secondary atom site location: difference Fourier map
*R*[*F*^2^ > 2σ(*F*^2^)] = 0.039	Hydrogen site location: mixed
*wR*(*F*^2^) = 0.073	H atoms treated by a mixture of independent and constrained refinement
*S* = 0.80	*w* = 1/[σ^2^(*F*_o_^2^) + (0.0216*P*)^2^] where *P* = (*F*_o_^2^ + 2*F*_c_^2^)/3
4432 reflections	(Δ/σ)_max_ = 0.001
231 parameters	Δρ_max_ = 0.16 e Å^−^^3^
0 restraints	Δρ_min_ = −0.14 e Å^−^^3^

### 7-Methoxy-5-methyl-2-(pyridin-4-yl)-11,12-dihydro-5H-5,11-methanobenzo[g][1,2,4]triazolo[1,5-c][1,3,5]oxadiazocine (II) Special details

**Table:**

Geometry. All esds (except the esd in the dihedral angle between two l.s. planes) are estimated using the full covariance matrix. The cell esds are taken into account individually in the estimation of esds in distances, angles and torsion angles; correlations between esds in cell parameters are only used when they are defined by crystal symmetry. An approximate (isotropic) treatment of cell esds is used for estimating esds involving l.s. planes.

### 7-Methoxy-5-methyl-2-(pyridin-4-yl)-11,12-dihydro-5H-5,11-methanobenzo[g][1,2,4]triazolo[1,5-c][1,3,5]oxadiazocine (II) Fractional atomic coordinates and isotropic or equivalent isotropic displacement parameters (Å^2^)

**Table:**

	*x*	*y*	*z*	*U*_iso_*/*U*_eq_	
O1	0.29552 (8)	1.00618 (8)	0.68647 (4)	0.0441 (3)	
O2	0.13197 (9)	0.92768 (9)	0.61850 (5)	0.0507 (3)	
N1	0.28301 (15)	1.24578 (13)	0.77525 (6)	0.0595 (4)	
H1A	0.2548 (17)	1.3017 (16)	0.7892 (9)	0.081 (7)*	
N2	0.22677 (12)	1.14540 (11)	0.86379 (6)	0.0491 (3)	
N3	0.29188 (11)	0.98503 (10)	0.82932 (5)	0.0453 (3)	
N4	0.31364 (11)	1.06389 (11)	0.78728 (6)	0.0455 (3)	
N5	0.13813 (13)	0.89571 (14)	1.04161 (7)	0.0604 (4)	
C1	0.13984 (16)	1.25155 (14)	0.64423 (8)	0.0553 (5)	
H1	0.144023	1.324469	0.649710	0.066*	
C2	0.05311 (15)	1.20940 (16)	0.60829 (9)	0.0625 (5)	
H2	−0.001899	1.253997	0.589972	0.075*	
C3	0.04590 (14)	1.10106 (15)	0.59873 (8)	0.0531 (5)	
H3	−0.013911	1.073106	0.574462	0.064*	
C4	0.12779 (13)	1.03541 (13)	0.62538 (7)	0.0425 (4)	
C5	0.21547 (13)	1.07829 (12)	0.66266 (6)	0.0403 (4)	
C6	0.22189 (14)	1.18600 (12)	0.67265 (7)	0.0437 (4)	
C7	0.31960 (15)	1.23155 (13)	0.71187 (7)	0.0518 (5)	
H7	0.345007	1.299766	0.695115	0.062*	
C8	0.42279 (14)	1.15486 (13)	0.71098 (8)	0.0528 (4)	
H8A	0.455901	1.150691	0.670365	0.063*	
H8B	0.484397	1.178879	0.738475	0.063*	
C9	0.37794 (13)	1.04721 (13)	0.73060 (7)	0.0436 (4)	
C10	0.47358 (13)	0.96458 (14)	0.73722 (7)	0.0548 (5)	
H10A	0.531162	0.988226	0.766340	0.082*	
H10B	0.438977	0.899338	0.750878	0.082*	
H10C	0.511468	0.953579	0.698666	0.082*	
C11	0.27338 (14)	1.15751 (13)	0.80925 (7)	0.0465 (4)	
C12	0.24111 (13)	1.03928 (13)	0.87389 (7)	0.0439 (4)	
C13	0.20625 (13)	0.98919 (13)	0.93123 (7)	0.0441 (4)	
C14	0.16193 (14)	1.04894 (15)	0.97868 (7)	0.0545 (5)	
H14	0.153807	1.121901	0.974754	0.065*	
C15	0.12990 (15)	0.99893 (17)	1.03192 (8)	0.0610 (5)	
H15	0.100395	1.040698	1.063278	0.073*	
C16	0.18286 (15)	0.84001 (17)	0.99569 (8)	0.0589 (5)	
H16	0.191250	0.767376	1.001142	0.071*	
C17	0.21758 (14)	0.88170 (13)	0.94085 (8)	0.0527 (4)	
H17	0.248277	0.838119	0.910630	0.063*	
C18	0.04328 (16)	0.87855 (15)	0.58270 (9)	0.0687 (6)	
H18A	0.054849	0.803252	0.583059	0.103*	
H18B	0.048312	0.904149	0.541693	0.103*	
H18C	−0.033421	0.894966	0.599078	0.103*	

### 7-Methoxy-5-methyl-2-(pyridin-4-yl)-11,12-dihydro-5H-5,11-methanobenzo[g][1,2,4]triazolo[1,5-c][1,3,5]oxadiazocine (II) Atomic displacement parameters (Å^2^)

**Table:**

	*U*^11^	*U*^22^	*U*^33^	*U*^12^	*U*^13^	*U*^23^
O1	0.0497 (6)	0.0416 (7)	0.0411 (6)	0.0029 (5)	−0.0089 (5)	−0.0003 (5)
O2	0.0551 (7)	0.0467 (8)	0.0502 (7)	−0.0023 (6)	−0.0106 (6)	−0.0008 (6)
N1	0.0996 (13)	0.0358 (10)	0.0432 (8)	0.0024 (9)	0.0045 (8)	−0.0013 (8)
N2	0.0637 (9)	0.0436 (9)	0.0399 (8)	−0.0009 (7)	0.0013 (7)	0.0009 (7)
N3	0.0543 (8)	0.0409 (8)	0.0407 (7)	−0.0029 (7)	−0.0002 (7)	0.0032 (7)
N4	0.0598 (8)	0.0372 (8)	0.0395 (7)	−0.0011 (6)	0.0015 (6)	0.0025 (7)
N5	0.0608 (10)	0.0736 (12)	0.0466 (9)	−0.0055 (8)	−0.0015 (8)	0.0085 (9)
C1	0.0714 (11)	0.0424 (11)	0.0521 (10)	0.0062 (10)	0.0014 (10)	0.0024 (9)
C2	0.0645 (12)	0.0555 (13)	0.0674 (13)	0.0164 (10)	−0.0051 (11)	0.0123 (11)
C3	0.0497 (10)	0.0591 (13)	0.0506 (11)	0.0030 (9)	−0.0065 (9)	0.0065 (10)
C4	0.0467 (9)	0.0406 (11)	0.0401 (9)	0.0008 (8)	0.0027 (8)	0.0030 (8)
C5	0.0440 (9)	0.0399 (10)	0.0368 (8)	0.0035 (8)	0.0021 (7)	0.0045 (8)
C6	0.0546 (10)	0.0398 (10)	0.0367 (9)	−0.0001 (8)	0.0040 (8)	0.0047 (8)
C7	0.0702 (11)	0.0413 (11)	0.0439 (10)	−0.0078 (9)	0.0021 (9)	0.0046 (9)
C8	0.0561 (10)	0.0547 (11)	0.0474 (10)	−0.0123 (9)	−0.0010 (8)	0.0012 (9)
C9	0.0470 (9)	0.0455 (10)	0.0383 (9)	−0.0034 (8)	−0.0031 (8)	−0.0013 (8)
C10	0.0514 (10)	0.0635 (12)	0.0495 (10)	0.0068 (9)	−0.0091 (8)	−0.0024 (9)
C11	0.0626 (11)	0.0357 (10)	0.0412 (9)	−0.0013 (8)	−0.0047 (8)	−0.0011 (9)
C12	0.0464 (9)	0.0420 (10)	0.0433 (9)	−0.0034 (7)	−0.0034 (7)	−0.0004 (8)
C13	0.0437 (8)	0.0465 (11)	0.0421 (9)	−0.0062 (8)	−0.0027 (7)	−0.0009 (8)
C14	0.0634 (11)	0.0524 (12)	0.0477 (10)	−0.0035 (9)	0.0001 (8)	−0.0016 (10)
C15	0.0673 (12)	0.0705 (15)	0.0451 (11)	−0.0039 (11)	0.0003 (9)	−0.0036 (11)
C16	0.0628 (11)	0.0551 (12)	0.0588 (12)	−0.0040 (10)	−0.0003 (10)	0.0117 (10)
C17	0.0587 (11)	0.0476 (11)	0.0518 (10)	−0.0046 (9)	0.0050 (9)	0.0004 (9)
C18	0.0694 (12)	0.0669 (14)	0.0697 (13)	−0.0007 (10)	−0.0176 (10)	−0.0199 (11)

### 7-Methoxy-5-methyl-2-(pyridin-4-yl)-11,12-dihydro-5H-5,11-methanobenzo[g][1,2,4]triazolo[1,5-c][1,3,5]oxadiazocine (II) Geometric parameters (Å, º)

**Table:**

O1—C5	1.3854 (16)	C5—C6	1.380 (2)
O1—C9	1.4408 (17)	C6—C7	1.513 (2)
O2—C4	1.3698 (18)	C7—C8	1.514 (2)
O2—C18	1.4165 (18)	C7—H7	0.9800
N1—C11	1.347 (2)	C8—C9	1.514 (2)
N1—C7	1.465 (2)	C8—H8A	0.9700
N1—H1A	0.83 (2)	C8—H8B	0.9700
N2—C11	1.3190 (19)	C9—C10	1.508 (2)
N2—C12	1.368 (2)	C10—H10A	0.9600
N3—C12	1.3263 (18)	C10—H10B	0.9600
N3—N4	1.3812 (17)	C10—H10C	0.9600
N4—C11	1.3557 (19)	C12—C13	1.465 (2)
N4—C9	1.4580 (19)	C13—C17	1.380 (2)
N5—C15	1.324 (2)	C13—C14	1.382 (2)
N5—C16	1.330 (2)	C14—C15	1.379 (2)
C1—C2	1.366 (2)	C14—H14	0.9300
C1—C6	1.390 (2)	C15—H15	0.9300
C1—H1	0.9300	C16—C17	1.374 (2)
C2—C3	1.387 (2)	C16—H16	0.9300
C2—H2	0.9300	C17—H17	0.9300
C3—C4	1.373 (2)	C18—H18A	0.9600
C3—H3	0.9300	C18—H18B	0.9600
C4—C5	1.395 (2)	C18—H18C	0.9600
			
C5—O1—C9	116.04 (12)	H8A—C8—H8B	108.4
C4—O2—C18	118.18 (13)	O1—C9—N4	107.90 (11)
C11—N1—C7	116.73 (15)	O1—C9—C10	106.13 (13)
C11—N1—H1A	117.7 (14)	N4—C9—C10	111.92 (13)
C7—N1—H1A	124.2 (14)	O1—C9—C8	110.29 (12)
C11—N2—C12	102.37 (14)	N4—C9—C8	106.29 (13)
C12—N3—N4	101.50 (12)	C10—C9—C8	114.20 (13)
C11—N4—N3	109.31 (12)	C9—C10—H10A	109.5
C11—N4—C9	126.70 (13)	C9—C10—H10B	109.5
N3—N4—C9	123.85 (13)	H10A—C10—H10B	109.5
C15—N5—C16	115.15 (16)	C9—C10—H10C	109.5
C2—C1—C6	120.34 (17)	H10A—C10—H10C	109.5
C2—C1—H1	119.8	H10B—C10—H10C	109.5
C6—C1—H1	119.8	N2—C11—N1	129.30 (16)
C1—C2—C3	120.96 (17)	N2—C11—N4	110.90 (14)
C1—C2—H2	119.5	N1—C11—N4	119.79 (15)
C3—C2—H2	119.5	N3—C12—N2	115.92 (14)
C4—C3—C2	119.44 (17)	N3—C12—C13	121.98 (15)
C4—C3—H3	120.3	N2—C12—C13	122.07 (15)
C2—C3—H3	120.3	C17—C13—C14	117.07 (15)
O2—C4—C3	125.16 (15)	C17—C13—C12	122.13 (15)
O2—C4—C5	115.23 (14)	C14—C13—C12	120.79 (15)
C3—C4—C5	119.61 (15)	C15—C14—C13	119.10 (17)
C6—C5—O1	123.62 (14)	C15—C14—H14	120.4
C6—C5—C4	120.92 (14)	C13—C14—H14	120.4
O1—C5—C4	115.43 (13)	N5—C15—C14	124.69 (18)
C5—C6—C1	118.71 (15)	N5—C15—H15	117.7
C5—C6—C7	120.25 (14)	C14—C15—H15	117.7
C1—C6—C7	121.00 (15)	N5—C16—C17	124.92 (19)
N1—C7—C6	112.58 (14)	N5—C16—H16	117.5
N1—C7—C8	107.90 (14)	C17—C16—H16	117.5
C6—C7—C8	108.02 (14)	C16—C17—C13	119.04 (17)
N1—C7—H7	109.4	C16—C17—H17	120.5
C6—C7—H7	109.4	C13—C17—H17	120.5
C8—C7—H7	109.4	O2—C18—H18A	109.5
C9—C8—C7	108.29 (13)	O2—C18—H18B	109.5
C9—C8—H8A	110.0	H18A—C18—H18B	109.5
C7—C8—H8A	110.0	O2—C18—H18C	109.5
C9—C8—H8B	110.0	H18A—C18—H18C	109.5
C7—C8—H8B	110.0	H18B—C18—H18C	109.5
			
C12—N3—N4—C11	−0.82 (15)	N3—N4—C9—O1	83.17 (16)
C12—N3—N4—C9	175.09 (13)	C11—N4—C9—C10	141.94 (15)
C6—C1—C2—C3	−0.9 (3)	N3—N4—C9—C10	−33.24 (19)
C1—C2—C3—C4	−0.6 (3)	C11—N4—C9—C8	16.63 (19)
C18—O2—C4—C3	−2.0 (2)	N3—N4—C9—C8	−158.54 (13)
C18—O2—C4—C5	178.11 (13)	C7—C8—C9—O1	65.73 (16)
C2—C3—C4—O2	−178.52 (15)	C7—C8—C9—N4	−50.97 (16)
C2—C3—C4—C5	1.3 (2)	C7—C8—C9—C10	−174.87 (13)
C9—O1—C5—C6	9.08 (19)	C12—N2—C11—N1	179.12 (17)
C9—O1—C5—C4	−172.98 (12)	C12—N2—C11—N4	0.13 (17)
O2—C4—C5—C6	179.17 (14)	C7—N1—C11—N2	−169.22 (16)
C3—C4—C5—C6	−0.7 (2)	C7—N1—C11—N4	9.7 (2)
O2—C4—C5—O1	1.17 (19)	N3—N4—C11—N2	0.44 (18)
C3—C4—C5—O1	−178.69 (13)	C9—N4—C11—N2	−175.31 (13)
O1—C5—C6—C1	177.12 (13)	N3—N4—C11—N1	−178.66 (14)
C4—C5—C6—C1	−0.7 (2)	C9—N4—C11—N1	5.6 (2)
O1—C5—C6—C7	−0.5 (2)	N4—N3—C12—N2	0.97 (17)
C4—C5—C6—C7	−178.34 (13)	N4—N3—C12—C13	−177.03 (13)
C2—C1—C6—C5	1.5 (2)	C11—N2—C12—N3	−0.73 (18)
C2—C1—C6—C7	179.10 (15)	C11—N2—C12—C13	177.26 (14)
C11—N1—C7—C6	73.9 (2)	N3—C12—C13—C17	−4.0 (2)
C11—N1—C7—C8	−45.3 (2)	N2—C12—C13—C17	178.14 (15)
C5—C6—C7—N1	−94.62 (18)	N3—C12—C13—C14	174.98 (14)
C1—C6—C7—N1	87.80 (19)	N2—C12—C13—C14	−2.9 (2)
C5—C6—C7—C8	24.4 (2)	C17—C13—C14—C15	−1.1 (2)
C1—C6—C7—C8	−153.16 (15)	C12—C13—C14—C15	179.90 (15)
N1—C7—C8—C9	67.08 (17)	C16—N5—C15—C14	1.2 (3)
C6—C7—C8—C9	−54.88 (16)	C13—C14—C15—N5	−0.1 (3)
C5—O1—C9—N4	74.33 (15)	C15—N5—C16—C17	−1.1 (3)
C5—O1—C9—C10	−165.55 (12)	N5—C16—C17—C13	0.0 (3)
C5—O1—C9—C8	−41.36 (16)	C14—C13—C17—C16	1.1 (2)
C11—N4—C9—O1	−101.66 (17)	C12—C13—C17—C16	−179.85 (14)

### 7-Methoxy-5-methyl-2-(pyridin-4-yl)-11,12-dihydro-5H-5,11-methanobenzo[g][1,2,4]triazolo[1,5-c][1,3,5]oxadiazocine (II) Hydrogen-bond geometry (Å, º)

**Table:**

*D*—H···*A*	*D*—H	H···*A*	*D*···*A*	*D*—H···*A*
N1—H1*A*···N3^i^	0.83 (2)	2.53 (2)	3.356 (2)	169 (2)
C1—H1···O1^i^	0.93	2.53	3.426 (2)	163
C7—H7···O2^i^	0.98	2.35	3.264 (2)	155

Symmetry code: (i) −*x*+1/2, *y*+1/2, *z*.
